# Field evaluation of HRP2 and pan pLDH-based immunochromatographic assay in therapeutic monitoring of uncomplicated falciparum malaria in Myanmar

**DOI:** 10.1186/1475-2875-12-123

**Published:** 2013-04-11

**Authors:** Myat H Nyunt, Myat P Kyaw, Kyu K Win, Khin M Myint, Khin M Nyunt

**Affiliations:** 1 Department of Medical Research, (Lower Myanmar), Republic of the Union of Myanmar; 2 University of Medicine, Yangon, Republic of the Union of Myanmar; 3 District Health Department, Myawaddy District, Myawaddy, Republic of the Union of Myanmar

**Keywords:** Malaria, RDT, Treatment monitoring, HRP2, pLDH

## Abstract

**Background:**

Malaria rapid diagnostic tests (RDT) are used for diagnostic purpose in malaria-endemic areas where reliable microscopy is not available. Persistence of the antigenaemia causes over-diagnosis and may limit the usefulness of the RDT in monitoring treatment. In this study, the usefulness of histidine-rich protein-2 (HRP2) and pan-specific or species-specific *Plasmodium* lactate dehydrogenase (pLDH) in treatment monitoring of uncomplicated falciparum malaria was carried out in an endemic setting in Myanmar.

**Methods:**

A prospective longitudinal, single-arm, cohort study was done by microscopy to confirm *Plasmodium falciparum* mono-infected cases. After direct treatment with an artemether-lumefantrine combination, patients were followed up on day 3, 7, 14, 21, 28 and any other day of recurrent fever. Blood film examination and RDT were carried out on day 0 and all follow-up days.

**Results:**

Out of 77 recruited falciparum cases, 63 became adequate clinical and parasitological response (ACPR) cases, and 60.3% of them were still positive for HRP2 up to day 28. Eleven out of 12 treatment failure cases (91.6%) were detected by pan pLDH. The mean duration required to become negative of HRP2 was 20 days (SD ± 6.03) and that of pan pLDH was six days with or without gametocytes and 3.7 days without gametocytes.

**Conclusion:**

Although treatment monitoring cannot be performed by HRP2, it can be assessed by pan pLDH-based assay after day 3 if a gametocidal drug has been administered and after day 7 if the presence of gametocytes was not excluded. The pan pLDH-based assay was a suitable test to monitor the treatment response of uncomplicated falciparum malaria patients.

## Background

The World Health Organization (WHO) recommends that malaria case diagnosis and management should be parasite-based and microscopy is still the cornerstone of diagnosis and remains the recommended method for therapeutic monitoring [1,2]. However, the staining process for malaria microscopy may take up to 60 minutes and is labour-intensive. Interpretation requires expertise and sequestered parasites in the deep capillaries cannot be detected by microscopy [3,4]. Also required is well-maintained equipment, a well-executed quality assurance system and trained staff.

Many new technologies, including immunochromatographic tests, appear to overcome the limitation of microscopy [5]. The test can be used easily as point of care at bedside or under field conditions at the peripheral level where reliable microscopy is not available [4,5]. It contains bound antibodies to specific antigens such as histidine-rich protein-2 (HRP2) (specific to *Plasmodium falciparum*), pan-specific or species-specific *Plasmodium* lactate dehydrogenase (pLDH) or aldolase, which is specific to all the major *Plasmodium* species [6]. The HRP2 is a water-soluble protein produced by trophozoites and young (but not mature) gametocytes of *P. falciparum*[7]. The pLDH [8] is produced by asexual and sexual stages (gametocytes) of malaria parasites. Test kits currently available detect pLDH from all *Plasmodium* species that infect humans [5,7].

Palmer *et al.* (1999) study showed that the immunochromatographic assay may be useful in post-treatment monitoring of malaria [9]. However, HRP2 may persist up to two weeks after chemotherapy [4,7] and it may be depend on the persistence viable asexual stage parasitaemia below the detection limit of microscopy [10], type of monoclonal antibody (IgG or IgM) used in the test [11], type of anti-malarial drugs used to treat the patient [12] and strain-specific differences in the antigen antibody interaction [13]. It is not adequate to give a definite conclusion because of the limited number of studies on detection of antigen by these rapid assays under field conditions up to 28 days’ follow up. In this study, HRP2 and pan pLDH-based immunochromatographic cassette assay was used for the first time to evaluate the role of these tests in post-treatment monitoring, according to WHO protocol for the assessment and monitoring of anti-malarial drug efficacy for the treatment of uncomplicated falciparum malaria under field conditions for up to 28 days’ follow up [2,14].

## Methods

### Areas of study

This prospective longitudinal, single-arm cohort study was done in Myanmar-Thailand border areas, Myawaddy Township, Kayin State from October to December 2010. The Township health profile showed that malaria was the leading cause of morbidity and mortality in this area from 2007 to 2009 and is defined as a hyper-endemic area [15]. The Myanmar-Thailand border is also one of the suspected loci for emerging drug resistance to falciparum malaria.

### Patients in the study

Sample size is calculated according to the anticipated proportion table [16] and 73 patients were included in the study, assuming that anticipated population proportion of clinical failures to artemether-lumefantrine 5%, confidence level of 95%, and precision of 10%.

Patients were recruited to the study were at least six years old, had mono-infection with microscopy confirmed *P. falciparum* (parasitaemia, 500–100,000 asexual forms per μl), axillary temperature ≥37.5°C or history of fever within previous 24 hours, ability to swallow oral medication and ability and willingness to comply with the study protocol for the duration of the study and to comply with the study visit schedule. Any person who showed presence of signs and symptoms of severe and complicated falciparum malaria according to current WHO definitions [2], mixed *Plasmodium* species, or other species of *Plasmodium,* presence of severe malnutrition, presence of febrile conditions due to diseases other than malaria, and pregnancy or lactating mothers, were excluded from the study.

### Ethical consideration

The study was approved by the ethical committee from Department of Medical Research (Lower Myanmar) and University of Medicine 1, Yangon. Written informed consents were taken from all the participants.

### Screening and enrolment procedures

The participants were screened by peripheral blood smear stained by 10% Giemsa and examined by microscopy. Individuals who met the inclusion criteria were enrolled, tested by HRP2 and panpLDH based RDT, thick and thin films examination for 3% Giemsa and treated on site with artemether-lumefantrine (Coartem®). The patients involved were monitored for a period of 28 days according to the scheduled visits.

### Microscopic blood examination

The microscopic blood film examinations were done according to WHO recommendations described in “Methods for surveillance of anti-malarial drug efficacy” [2]. Thick and thin blood films for parasite count were obtained and examined at screenings on day 0, 1, 2, 3, 7, 14, 21 and 28 or on any other day if the patient spontaneously returned and parasitological reassessment was required. All the slides were counter-checked by two independent, qualified microscopists and if a result showed more than 20% discrepancy, an expert microscopist was requested to check the slide and the average count of the two similar results was recorded.

### Immunochromatographic cassette assay test

In this study, malaria antigen (HRP2 and pan pLDH based) Pf/Pan immunochromatographic test kit (SD® Bioline, Cat No 05FK60, Lot No 090007, Expiry date 2011.06.08, Standard Diagnostics Inc, Korea) was used. Test procedure was done according to manufacturer’s instruction. Briefly, blood sample (5 μl) was taken from finger tip by a capillary pipette, and the open end immersed in the blood drop and then gently released for the pressure to draw blood into the capillary pipette to sample well. Four drops of assay diluents were added to the assay diluent well. The result was read after 15 minutes; never after 30 minutes to avoid false result. Therapeutic evaluation of the patients was not interfered by the result of this test. Band intensity of the result was noted as follows: 0 no band (negative), 1+ faint band, but clearly visible (positive), 2+ medium intensity bands, stronger than 1+ but less than control band (positive), 3+ equal or stronger than the control band (positive).

### Quality control of the test kit

The test kits were stored in a temperature and humidity control room at the Quality Control Laboratory for malaria rapid diagnosis test (RDT), Department of Medical Research (Lower Myanmar). In the field, the test kits were stored at the recommended temperature and humidity. Temperature and humidity were recorded three times a day, i e, 6 am, 12 noon and 6 pm by using a thermo hygrometer to ensure the recommended temperature and humidity. After the study, 10 test kits from the field were randomly selected and tested with the quality control blood samples that were prepared according to the methods manual for laboratory quality control testing of malaria RDT to check the validity of the assay kits [17].

### Data analysis

After the study was completed, data were entered onto a database using double independent data entry by using Microsoft Excel and SPSS software version 16. Sensitivity, specificity, positive predictive value, negative predictive values were calculated as blood film examination was gold standard. Sensitivity is the probability (percentage) that patients with the infection (determined by the result of the reference or ‘gold standard’ test, blood film examination by microscopy) will have a positive result using the test under evaluation.

## Results

A total of 844 patients were screened by active case detection and malaria parasites were detected in 40.9% of the screening slides (n = 346). The 63.2% (219 cases) of the microscopy positive cases were *Plasmodium vivax*; 29.4% (102 cases) were *P. falciparum;* and, 7.2% (25 cases) were mixed infection, i.e, *P. falciparum* and *P. vivax.*

**Figure 1:**
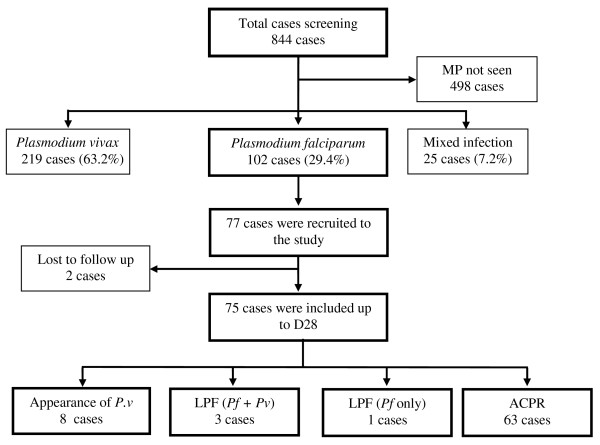
**Screening algorithm of the study cases.** MP = malaria parasite, *Pv = Plasmodium vivax, Pf = Plasmodium falciparum,* LPF = late parasitological failure, ACPR = adequate clinical and parasitological response.

Of the *P. falciparum* mono-infection, 77 participants were recruited for the study. The screening algorithm of study cases is shown in Figure 1. Baseline and parasitological characteristics of the study patients are shown in Table 1. The data was analysed first for treatment failure patients and then adequate clinical and parasitological response (ACPR) cases.

**Table 1:** Baseline and parasitological characteristics of the study populations

**Baseline characteristics on day 0 (N = 77)**	
Mean age in years	23.83
Range of age in years (minimum, maximum)	6–56
Sex ratio (male/female)	2.08 (52/25)
Mean weight in kg	41.35
Mean height in cm	146.57
Mean temperature (°C)	38.95
History of fever within 24 hours (%)	100
Haemoglobin level (mean, range)	10.7 (6.5–15)
Parasitological characteristics on day 0 (N = 77)	
Initial asexual parasitaemia density range (parasites/μl)	580–96000
Mean of initial asexual parasitaemia density (parasites/μl)	15672
Initial *Plasmodium falciparum* gametocyte carrying cases	22.08% (17)
Range of gametocyte density (per ul)	21–4316

### Early detection of the treatment failure

The result of HRP2 for all the treatment failure patients of *P. falciparum* (n = 4) was positive on day of failure*.* However, long-term persistence of HRP2 was observed before day of treatment failure in all of the cases. Persistence positive of the HRP2 was observed up to the day of failure. It was difficult to differentiate between the persistence of antigen from newly emergent antigen. Their band intensity was increased in two failure cases but was the same as the previous schedule visit in the other two.

In 12 parasite-reappeared cases, there was no persistence positivity of pan pLDH results. Band intensity of the pan pLDH was decreased on subsequent follow-up days, a feature which was parallel to the microscopy result. The reappearance of the band of the pan pLDH was observed in all parasite-reappeared cases regardless of the species, except in only one case in which parasite density was low (31 per μl of blood) (Table 2).

**Table 2:** Parasite species, density and re-appearance day with the result of the HRP2 and pLDH in parasite reappearance cases

**No. of cases**	**Parasite re-appearance day**	**Blood film examination**		**RDT result**		
		***Pf *(/ul)**	***Pv *(/ul)**	**Control**	**HRP2**	**Pan pLDH**
1	D21	8415	0	3+	3+	2+
2	D21	855	70	3+	3+	1+
3	D21	0	82	3+	0+	1+
4	D26	1081	2513	3+	2+	3+
5	D28	0	762	3+	1+	2+
6	D28	110	527	3+	1+	3+
7	D28	0	2535	3+	0	3+
8	D28	0	12891	3+	1+	3+
9	D28	0	153	3+	1+	1+
10	D28	0	3339	3+	2+	3+
11	D28	0	3550	3+	1+	1+
12	D28	0	31	3+	0	0

### Sensitivity, specificity, positive predictive value and negative predictive value of the HRP2 and pLDH

In this study, a total of 453 HRP2 and pan pLDH combo tests were done in 77 uncomplicated falciparum malaria patients and no invalid result was observed. The sensitivity, specificity, positive predictive value and negative predictive value of the assay were calculated on day 0 and subsequent follow-up visits up to day 28.

The sensitivity of the HRP2 was higher than that of the pan pLDH in all follow-up visits. However, pLDH was more specific than HRP2. The HRP2 had 100% sensitivity in almost all of the testing times, i.e, day 0 through day 28 although its specificity was low (1.32–39.44%) (Table 3).

**Table 3:** **Sensitivity, specificity, positive predictive value and negative predictive value of the HRP2 and pan pLDH-based test (*Plasmodium falciparum *gametocytaemia was considered negative for *P. falciparum*)**

**Day**	**Sensitivity (95% CI)**		**Specificity (95% CI)**		**Positive predictive value**		**Negative predictive value**	
	**HRP2**	**pLDH**	**HRP2**	**pLDH**	**HRP2**	**pLDH**	**HRP2**	**pLDH**
D0	100	100	NR^2^	NR^2^	100	100	NR^3^	NR^3^
	(95.3–100%)	(95.3–100%)			(95.3–100%)	(95.3–100%)		
D3	100	50	0	73.97	5.19	9.52	NR^3^	96.43
	(39.7–100%)	(6.8–93.2%)	(0.0–4.9%)	(62.4–83.6%)	(1.4–12.8%)	(1.2–30.4%)		(87.7–99.6%)
D7	NR^1^	NR^1^	1.32	85.53	0	0	100	100
			(0.1–7.1%)	(75.6–92.6%)	(0–4.8%)	(0–28.5%)	(2.5–100%)	(94.5–100%)
D14	NR^1^	NR^1^	9.33	93.33	0	0	100	100
			(3.8–18.3%)	(85.1–97.8%)	(0–5.3%)	(0–52.2%)	(59.0–100%)	(94.9–100%)
D21	100	100	27.40	93.06	3.64	37.50	100	100
	(15.8–100%)	(29.2–100%)	(17.6–39.1%)	(84.5–97.7%)	(0.4–12.5%)	(8.5–75.5%)	(83.2–100%)	(94.6–100%)
D28	100	88.89	39.44	96.87	4.44	80	100	98.41
	(15.8–100%)	(51.8–99.7%)	(29.0–51.8%)	(89.2–99.6%)	(0.5–15.2%)	(44.4–97.5%)	(87.7–100%)	(91.5–99.9%)

NR = Not relevant.

^1^ Sensitivity was not calculated because of no true positive.

^2^ Specificity was not calculated because of no true negative.

^3^ NPV was not calculated because of no true negative.

The pLDH had high specificity (73.97–96.87%). The specificity was increased to 87.10–100% if *P. falciparum* gametocytaemia was considered positive for *P. falciparum* but it was not significant (*χ*^2^ = 0.756, p = 0.9443, 95% CI). However, if *P. falciparum* gametocytaemia was considered positive for *P. falciparum,* the sensitivity of the pan pLDH was significantly increased (*χ*^2^ = 155.35, p < 0.0001, 95% CI)*.*

**Figure 2:**
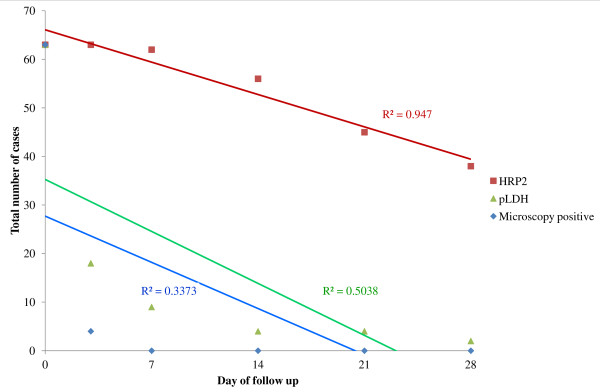
Comparison of HRP2 and pLDH result with microscopy in each of the follow-up days in all ACPR cases.

The results of pan pLDH were parallel to the microscopy result on day 0 and all of the follow-up days (*χ*^2^ = 2.853, p = 0.7227, 95% CI) in all ACPR cases. However, positive results of HRP2 were significantly higher than of microscopy (*χ*^2^ = 92.936, p <0.0001, 95% CI) as shown in Figure 2.

### Persistence of HRP2 and pan pLDH in ACPR cases

Among 63 ACPR cases, 60.32% were still positive for HRP2 assay up to day 28. Three of the HRP2 positive cases showed 3+ band intensity on day 28. Although, the continuous decreasing of the band intensity of HRP2 was observed in most of the ACPR cases, equal band intensity, i.e, 3+, as occurred on the initial day, could not exclude the resolving infection.

Regarding the pan pLDH, only 3.17% (n = 2) showed 1+ band intensity on day 28. Without gametocytes, only five cases of false positive were observed on day 3. There was no case of more than +1 band intensity in all of the cases, with or without sexual parasite, on and after day 3.

### Persistence of antigenaemia after treatment with the initial parasite count

**Figure 3:**
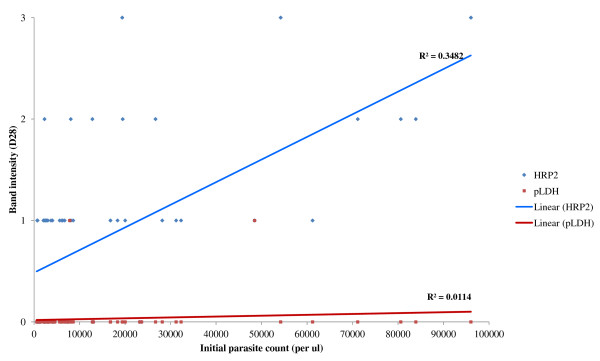
**Correlation of persistence of band intensity of HRP2 and pLDH with initial parasite count.** Higher initial parasite counts were correlated with the higher persistence of the band intensity in HRP2-based RDTs.

Long-term persistence of the false positive was directly correlated with the initial parasite count in ACPR cases (p = 0.000 for both HRP2 and pLDH). Initial parasite count was also correlated with the persistence of higher band intensity of HRP2 on day 28 as shown in Figure 3.

### Day required to become negative result of HRP2 and pan pLDH after treatment

Among the 63 ACPR cases, a total of 25 cases became HRP2 negative before day 28 and all 63 cases became pLDH negative within 28 days. Mean time to become negative result of HRP2 was 20 days (SD ± 6.03) and pLDH was six days (±6.31). If gametocytes were considered positive for *P. falciparum*, the mean time required to become negative result of HRP2 was 21 days (SD ± 6.41) and of pLDH was 3.7 days (SD ± 4.11).

### False positive of the HRP2 and pan pLDH

Day 3 persistence of asexual parasitaemia was detected in four patients. On day 3, 59 (94%) were HRP2 false positive. On day 7, 62 (98%) were HRP2 false positive. A total of 56 (89%) on day 14, 45 (71%) on day 21, and 38 (60%) on day 28 were still positive without asexual parasitaemia. If *P. falciparum* gametocytaemia was considered positive for *P. falciparum*, false positive of pLDH was less than 10%. However, false positive for HRP2 was more than 60% in all of the follow-up visits even if *P. falciparum* gametocytaemia was considered positive for *P. falciparum*.

## Discussion

The therapeutic monitoring and early detection of treatment failure is very important in management of malaria cases in the era of increasing drug resistant malaria. The ability of the test to monitor response to therapy is ideal in malaria-endemic areas, especially where drug resistant *P. falciparum* has been documented [18].

HRP2 persistence makes early detection of treatment failure difficult. The sudden increase of the band intensity of HRP2 on subsequent follow-up visits is an alert to further investigation to confirm treatment failure. However, without continuous monitoring of cases by the recording of band intensity, a definite conclusion cannot be reached of treatment failure. Moreover, being the HRP2 produced from *P. falciparum* only, it was useless in non-*P. falciparum* infection [11]. The pan pLDH can detect all treatment failure cases with a parasite count of more than 50 per μl. The pan pLDH can detect not only *P. falciparum* but also non-*P. falciparum* infection, but it cannot differentiate between the two. However, if the sexual stage parasites were still present on follow-up visits, and the pLDH test became positive, this can cause confusion in early detection of the treatment failure [19].

There were many studies focusing on the sensitivity of pan pLDH. However, the results were varied: one study in Uganda [20] found that its sensitivity was 95.6%, and a study in Madagascar [21] found pLDH sensitivity was 97%. In a study in Myanmar using CareStart®, two-line pan pLDH assay showed sensitivity of 94.7% [22]. In this study, sensitivity and positive predictive value of HRP2 and pan pLDH were 100% in all of the study cases on day 0. There was no false negative in both of the assay on day 0. Sensitivity of the HRP2 was 100% in all of the subsequent follow-up days up to day 28. The sensitivities of the pan pLDH assay were varied 50 to 100% in subsequent follow-up visits. The sensitivities were increased up to 80 to 100% if *P. falciparum* gametocytaemia was considered positive for *P. falciparum,* indicating that persistence gametocytaemia may cause positive result of pLDH. Therefore, it is necessary to follow the National Anti-malarial Treatment Guidelines and WHO recommendations, which mention to add a single dose of primaquine (0.75 mg/kg) as a gametocidal drug to artemisinin combination therapy for uncomplicated falciparum malaria [23].

The result of pan pLDH was parallel to that of microscopy. However, the results of HRP2 cause more false positives due to the long persistence of antigenaemia on subsequent follow-up days. The long-term persistence of HRP2 reduces its usefulness in monitoring the response to treatment. It was documented that during follow up after treatment, 98.2%, 94.6%, 92.0% and 73.5% of effectively treated children were still false-positive by RDT at day 14, 21, 28 and 35, respectively, and this antigenaemia could persist up to 35 day after treatment [24]. In this study, more than half of the ACPR cases were still HRP2 positive on day 28, three of whom were 3+ band intensity. Only 20 cases become HRP2 negative before day 28 and the mean time required to become HRP2 negative was 20 days. It was longer than that of pan pLDH, which was six days and it would be shortened down to 3.7 days if *P. falciparum* gametocytaemia was considered positive for *P. falciparum*. Other studies showed the median duration for pLDH to become negative was two days for CareStart® malaria tests and seven days for OptiMAL-IT® [25].

However, continuous reducing of the band intensity of HRP2 was observed in most of the ACPR patients. There were evidence of association of the band intensity and parasite count [26], a ‘plus system’ was used for band intensity in this study. Initial parasite count is directly correlated with the band intensity of the assay. Moreover, initial parasite count was correlated with the higher band intensity of the HRP2 test. Therefore, the band intensity of the HRP2 may be a useful tool to access treatment response in uncomplicated falciparum malaria cases.

The frequent occurrence of false positive results can lead to unnecessary treatment. This can have several negative outcomes, including clinicians inappropriately focusing on malaria, and not identifying the true cause of illness, and unnecessary exposure to anti-malarials. In some cases, the inappropriately treated patient may return with similar symptoms, leading the clinician to falsely report the presence of parasite drug resistance. This could lead to the clinician not trusting the efficacy of the first-line anti-malarial and consequently dispensing the second-line anti-malarial, increasing the cost of treatment and further delaying appropriate treatment [22]. The treatment failure cases may be due to recrudescense or re-infection (new infection). However, RDTs cannot differentiate recrudescence from re-infection among treatment failure cases as only molecular methods can differentiate. Moreover, RDTs are designated to detect malaria infection qualitatively and brand to band quality variation was common, which could affect the use of these tests.

## Conclusion

In this study, HRP2 had higher sensitivity than pan pLDH assay. However, specificity was higher in pan pLDH. The persistence of HRP2 up to day 28 in ACPR cases causes false positive results and this is the reason HRP2 is not fit for monitoring treatment response. The pan pLDH can also detect late parasitological failure and non-*P. falciparum* infection during the follow-up period. The persistence of pan pLDH was shorter in duration than that of HRP2. The meaningful result of pan pLDH can be observed in monitoring treatment response in uncomplicated falciparum malaria patients after day 3 of artemisinin combination therapy if the gametocidal drug was added according to the recommendation by WHO in 2010. Therefore, pLDH is useful for therapeutic monitoring of uncomplicated falciparum malaria patients.

## Competing interests

The authors declare that they have no competing interests.

## Authors’ contributions

MPK, KKW and MHN conceived and designed the study. MHN conducted the field work. KMN and KMM conducted data validation and management. All authors contributed during writing, and read and approved the manuscript.
